# Unveiling Intra-Clonal Diversity of Monkeypox Virus from Brazil’s First Outbreak Wave

**DOI:** 10.3390/v18010062

**Published:** 2025-12-31

**Authors:** Amanda Stéphanie Arantes Witt, João Victor Rodrigues Pessoa Carvalho, Izabela Mamede, Talita Emile Ribeiro Adelino, Felipe Campos de Melo Iani, Maurício Teixeira Lima, Thalita Souza Arantes, Denilson Eduardo Silva Cunha, Rodrigo Araújo Lima Rodrigues, Giliane de Souza Trindade, Erna Geessien Kroon, Nidia Esther Colquehuanca Arias, Glória Regina Franco, Jônatas Santos Abrahão

**Affiliations:** 1 Laboratório de Vírus, Departamento de Microbiologia, Instituto de Ciências Biológicas, Universidade Federal de Minas Gerais, Belo Horizonte 31270-901, Braziljvrodrigues934@gmail.com (J.V.R.P.C.); rodriguesral07@gmail.com (R.A.L.R.); nidiaestherarias@gmail.com (N.E.C.A.); 2 Laboratório de Genética Bioquímica, Departamento de Bioquímica, Instituto de Ciências Biológicas, Universidade Federal de Minas Gerais, Belo Horizonte 31270-901, Brazilgfrancoufmg@gmail.com (G.R.F.); 3 Serviço de Virologia e Riquetsioses, Instituto Octávio Magalhães, Fundação Ezequiel Dias, Belo Horizonte 30510-010, Brazil; 4 Instituto Nacional de Ciência e Tecnologia em Poxvírus, Belo Horizonte 31270-901, Brazil; 5 Centro de Microscopia, Universidade Federal de Minas Gerais, Belo Horizonte 31270-901, Brazildenilsonescunha@yahoo.com.br (D.E.S.C.)

**Keywords:** monkeypox virus, clone, phenotypic characterization, genome, SNVs, 2022 outbreak, MPOX

## Abstract

The monkeypox virus (MPXV) is an emerging zoonotic pathogen responsible for mpox, a disease characterized by some smallpox-like symptoms, typically mild but occasionally fatal. The largest mpox recorded global outbreak began in May 2022, with over 162,000 cases across 140 countries. Herein, we have analyzed the intra-clonal diversity of MPXV obtained from a single skin lesion sample from a male patient (June 2022). Three viral clones were obtained following phenotypic evaluation of MPXV lysis plaque characteristics over a three-course infection in BSC-40 cells. Unlike the vaccinia virus Western Reserve (VACV-WR) strain, MPXV clones did not produce comet-like structures, suggesting reduced extracellular enveloped virus (EEV) morphotype release, which is associated with viral dissemination. Whole-genome sequencing and assembly identified subtle differences among clones. Comparative genomic analyses, including synteny and single nucleotide variation (SNV) calling, revealed intra-clonal differences and divergence from clade I and II references, although the variety of mutations found did not reveal possible variations at the protein level. Altogether, these findings suggest that although similar, it is possible that distinct MPXV variants may circulate together and can be found in a single exanthematous lesion.

## 1. Introduction

The monkeypox virus (MPXV) was first described in 1958 (Copenhagen, Denmark) associated with captive cynomolgus monkeys (*Macaca fascicularis*) presenting with a smallpox-like infection [1]. The first human MPXV infection (mpox) was reported years later in the Democratic Republic of Congo in a 9-month-old patient diagnosed by the World Health Organization (WHO) Smallpox Reference Center (Moscow, Russia) [1,2]. Mpox symptoms are similar to smallpox but are typically mild, including fever, body chills, swollen lymph nodes, exhaustion, headache, adenopathy, and vesiculopustular rashes. The infection lasts from 3 to 17 days and, in severe cases, may lead to death [3].

Current viral taxonomy classifies poxviruses as belonging to the *Nucleocytoviricota* phylum, the *Pokkesviricetes* class, *Chitovirales* order, family *Poxviridae*. The monkeypox viruses have been classified into two genetic clades: clade I, prevalent in Central Africa (Congo Basin), and clade II, endemic to West Africa—based on geographic origin and endemicity. These clades are subdivided into lineages (clade I lineages 1–5 and clade IIa and IIb lineages) with distinct lethality rates [4,5,6]. Phylogenetically, the Clade IIb MPXV shares 99.6% nucleotide identity with Clade I and 99.7% nucleotide identity with Clade IIa [7]. MPXV genome consists of a double-stranded DNA strand ranging in size from 196 to 211 kilobases (Kbp), containing the information required to encode about 200 genes [8]. Interestingly, there seem to be some differences among genomic features when comparing both MPXV clades. For example, clade I is more uniform in terms of genome size than clade II. Additionally, one clade has genes that the other does not [9].

Historically, this zoonotic virus has been reported endemically in African countries since its discovery, with rare cases of mpox outside the continent until 2022, either caused by clade I, clade IIa, or clade IIb [7]. As of September 2025, the ongoing global epidemic of mpox that began in 2022 registers more than 162,000 confirmed cases in 140 countries. Of all the non-endemic regions where MPXV has circulated since May of 2022, the region of the Americas is the most affected, with the United States of America and Brazil being the countries with the most confirmed mpox reported cases (35,590 and 14,328 reported cases, respectively) (2022–2025) [3]. Moreover, the 2022 global mpox outbreak is linked to the emergence of the clade IIb MPXV viruses, which has also been previously associated with mpox outbreaks in Nigeria, the United Kingdom, Israel, and Sin39gapore [7,10,11].

Nevertheless, mpox has been considered a neglected disease due to its restricted geographical distribution until its global emergence in 2022. Given the proportions of the 2022 ongoing mpox multicontinental outbreak, MPXV has been studied through many distinct approaches that aimed to better understand various aspects of its biology. As usual, genomic surveillance has played a key role in our understanding of the diversity of MPXV following the 2022 mpox outbreak. However, it is worth noting that, since the beginning of the outbreak in 2022, most of the sequenced viral isolates have provided genomic sequences that reflect the characteristics of the sample as a whole. Therefore, further information about the genomic variation within the sample (i.e., intra-sample genomic diversity) remains poorly explored, creating a gap in our understanding of the MPXV genomic diversity that has yet to be filled.

In this context, the phenotypic characterization of MPXV lysis plaques in vitro provides insight into the variation in the virus within the same sample. As has been demonstrated for other orthopoxviruses (OPVs), such as the Vaccinia virus (VACV), OPV species can co-circulate as distinct genetic groups or viral populations that exhibit significant phenotypic differences, which may even contribute to viral evolution rate by genome recombination and viral diversity [12,13]. In light of the ongoing efforts to improve our understanding of MPXV biology following the unusual global outbreak in 2022, here we explore phenotypic and genomic features of MPXV clones obtained from a single exanthematous lesion. Our results suggest that different MPXV variants can be found in a single lesion, contributing to our understanding on MPXV IIb clade evolution.

## 2. Methods

### 2.1. Sample Description

The MPXV sample used in this study was obtained from a single genital lesion of a male patient admitted to a public health system hospital in Brazil in June 2022 [14]. The molecular diagnosis was performed by the Ezequiel Dias Foundation (FUNED), the reference laboratory for diagnosis of infectious diseases in Minas Gerais State, Brazil (Ethics Committee approval, CAAE: 62702222.6.0000.9507, SISGEN-AF47BB2) Part of the sample was submitted to DNA extraction, followed by PCR targeting MPXV, as described by CDC [15]. The molecular diagnosis by PCR confirmed that the patient was infected with MPXV. After PCR confirmation, 15 µL of the vesicular content of the lesion was sent to the Virus Laboratory at the Federal University of Minas Gerais (UFMG), Brazil, where it was processed for virus isolation and clarification. The sample was manipulated in a BSL-3 laboratory, following all institutional biosafety protocols. Five hundred microlitres of Dulbecco’s modified essential medium supplemented with 1% fetal calf serum was added to the vesicular content, homogenized, and then inoculated into a monolayer of Vero cells in a T75 culture flask for virus clarification. Vero cells were used for this stage of the viral manipulation process, as Vero cells are more permissive of OPV in vitro infection. Plaque-forming units (PFUs) were observed at 48 h post-infection (h.p.i.) under incubation at 37 °C and 5% CO_2_. Cells were collected by physical detachment using a sterile cell scraper, and a portion of this extract was re-inoculated into a new monolayer of Vero cells for confirmation and observation of PFU.

### 2.2. Plaque Phenotype Characterization Assay and Viral Clone Isolation

To identify possible phenotypic variation between different MPXV lysis plaques, three consecutive passages of infection, PFU observation, and collection were performed on BSC-40. Similarly to Vero, the BSC-40 is an excellent cell lineage for in vitro OPV infections. However, it is a better platform for evaluating the lysis plaque phenotype due to its slightly reduced size, which allows for more defined plaques. For phenotypic characterization of MPXV lysis plaques and clone isolation, BSC-40 cells at 95% confluence were used. For each infection, the virus sample was serially diluted in minimal essential medium (MEM) containing 0% fetal calf serum, after which the plates were incubated at 37 °C and 5% CO_2_ for one hour to allow adsorption. After adsorption, the virus inoculum was completely removed, and a semi-solid medium of MEM supplemented with 1% fetal calf serum and 0.5% agarose was carefully added to each well. The cells were then incubated at 37 °C and 5% CO_2_, and the PFUs were carefully observed using a Sunny Instruments SOP TOP ICX41 microscope at 40× and 100× magnification. The PFUs were selected after 48 h of incubation. The PFUs were selected based on characteristics such as approximate size, shape, and peripheral cellular aspects, as well as the presence or absence of cells and cellular debris within the PFU. The selected PFUs were carefully collected using 10 µL filtered pipette tips positioned vertically over the PFU and stored at −80 °C in MEM containing 10% fetal calf serum to maximize viral particle stability. This procedure was repeated for each of the three consecutive infections.

For the first of the three passages, we have used the clarified viral sample after serial dilution. PFU collected from the first infection were labeled, serially diluted, and used for the second infection. Similarly, PFU collected from the second infection were labeled, serially diluted, and used for the third and last infection. By the end of the three-course infection assay, the final PFUs were collected and determined as final viral clones. At last, MPXV clones were expanded into T75 flasks containing BSC-40 monolayer at 90% confluency and MEM 1% fetal calf serum under incubation at 37 °C and 5% CO_2_ until observation of cytopathic effect and formation of plaques. Cell monolayers were washed with PBS buffer, and collected with a sterile cell-scraper, and both pellet and supernatant were stored in cryotubes at −80 °C.

### 2.3. Comet-Forming Assay

We conducted a comet-forming assay in order to evaluate viral dissemination of MPXV clones through EEV liberation. For this assay, 6-well plates containing BSC-40 monolayers at 95% confluency were infected with each MPXV clone serially diluted in MEM 0% fetal calf serum, followed by adsorption at 37 °C and 5% CO_2_ for an hour. VACV-WR was included as a positive control for comet comparison. After the adsorption period, 2.5 mL MEM supplemented with 1% fetal calf serum was added per well, and plates were incubated at a slight incline for 72 h at 37 °C and 5% CO_2_. Subsequently, the cell monolayers were fixed with 3.7% formaldehyde and stained with 1% crystal violet solution to visualize plaques and any comet visualization. The stained monolayers were examined macroscopically and over a transilluminator to assess plaque morphology.

### 2.4. Viral DNA Extraction

Viral DNA was extracted from 200 µL of supernatant from each MPXV clone, previously expanded in BSC-40 cells and harvested as described in the Plaque phenotype characterization assay and viral clone isolation section. The extraction was performed using the High Pure Viral Nucleic Acid Kit (Roche 14781400) (Roche Diagnostics GmbH, Mannheim, Germany) following the manufacturer’s instructions. Briefly, samples were incubated with Binding Buffer, poly(A), and proteinase K at 72 °C for 10 min to lyse viral particles and degrade nucleases. The resulting lysates were then loaded onto the kit’s spin columns to facilitate nucleic acid binding to the silica membrane through a series of centrifugation and washing steps using the supplied Inhibitor Removal Buffer and Wash Buffer. Finally, the purified DNA was eluted in 50 µL of Elution Buffer. The extracted DNA samples were immediately stored at −20 °C for subsequent sequencing.

### 2.5. Viral Genome Sequencing, Gene Annotation, and SNV Calling

Viral DNA was amplified by MPV-specific multiplex PCR using tiling primers and Q5 High-Fidelity DNA Polymerase (New England Biolabs, Ipswich, MA, USA) as previously described by Guimarães et al. (2023), and sequenced on an Illumina MiSeq with normalized libraries loaded onto a 300-cycle according to the manufacturer’s instructions (Illumina, San Diego, CA, USA) [14]. Raw reads were trimmed with Trimmomatic (v 0.36.6) using the SLIDINGWINDOW filter [16]. Genome assemblies were generated with SPAdes v4.2.0 via Galaxy (tool spades/4.2.0+galaxy0), and reads and contigs were then mapped with Minimap2 v2.24 to the Clade IIb reference (NC_063383) [17,18,19]. Consensus sequences were aligned with MAFFT v7.450, visualized in Geneious Prime (v2024.1.2), and subsequently manually curated [20,21]. Assemblies are available at SRA under accession PX448464, PX448465 and PX448466.

The assembled genome for the isolates was aligned against the Zaire reference using MAFFT. CDS calling was performed using Prodigalv2.6.3 and the predicted ORFs were annotated following the workflow described by Carvalho et al. (2024) [22,23]. Briefly, we used Blastp (e-value < 10^−5^) against the NCBI nr database, followed by protein domain search using HHpred [24,25,26]. In case of results discordance, we used Interproscan and compiled the results to achieve the final gene annotation [27]. The genome and a gene identity comparison was performed using data matrixes from minimap2-2.30 for genomes and FastANI v1.34 for genes, that was then used as inputs for heatmaps, using the package pheatmap (kolde, 2025—https://github.com/raivokolde/pheatmap (accessed on 12 September 2025)) in R version 4.4.3 (R Core Team, 2025) [17,28,29].

To compare conservation among the genomic sequences, we used DigAllign 2.0, using the reference genomes for Clade I MPXV strain Zaire 1979-005 (GenBank accession: HM172544), and clade II MPXV 2022 (GenBank accession: NC_063383) reference viruses [30]. The order was chosen to better represent their similarity. SNV calling was performed using an in-house script available at https://github.com/iza-mcac/2025-10-Pox-Variant_calling (accessed on 12 September 2025), and the resulting variants were filtered to include only those associated with genes in Monkeypox virus [31]. In summary, we used Biostrings and tidyverse packages in R, where variants were annotated based on their position relative to coding regions in the reference genome. Custom functions classified SNVs as synonymous, non-synonymous, or stop-gained based on codon changes, while indels were categorized as frameshift or non-frameshift. Annotated variants were reshaped into a wide-format table for comparison across viral clones.

Additional analysis of the original sample sequenced by Guimarães et al. (2023) has been conducted in consideration of the inherent possibility of identifying in vitro-derived SNVs [14]. The raw reads obtained from the sequencing of the original clinical sample were aligned to the MPXV clade II reference genome (NC_063383.1). Each clonal SNV position identified previously was used to find and quantify by extraction the number of reads supporting the reference base and the alternate observed base on the original genome. The alternative allele frequency count was considered a parameter for clonal SNV validation as proven represented within the original sample.

### 2.6. Phylogenetic Reconstruction

A dataset of 284 genomes was retrieved from GISAID (accessed on 10 October 2025) [32]. After re-evaluating sequences and metadata, we excluded entries with anomalous insertions or excess mutations, yielding 248 public genomes plus 3 clonal genomes, alongside the MPV RefSeqs for Clade I (NC_003310) and Clade II (NC_063383). Genomes were aligned with MAFFT v7.450, and the alignment was trimmed with ClipKIT v0.1.0 in smart-gappy model [21,33]. Maximum-likelihood tree was inferred with IQ-TREE 2 v2.4.0 using 1000 bootstrap replicates [34]. The best-fit substitution model was selected by ModelFinder [35]. Trees were visualized and edited in iTOL v7 [36].

### 2.7. Structural Comparisons

Structural modeling was performed using SwissModel web server and AlphaFold2, via ColabFold v1.5.5, using pdb100 as a database for templates [37,38]. The models generated by AlphaFold were used in FoldSeek to search for homologous structures [39]. Predicted domains were characterized using InterProScan 106.0 and pHMMER 3.3.2 web servers [27,40]. The Gibbs free energy change values for the mutated amino acids were calculated using the MAESTRO 1.2.35 web server [41]. Physico-chemical parameters were measured using Ramachandran plots, generated in RamplotR online tool (https://bioit.shinyapps.io/RamplotR/ (accessed on 12 September 2025)), and ProtParam [42].

## 3. Results

### 3.1. MPXV Lysis Plaque Phenotype Variation and Clone Selection

In this study, we have evaluated MPXV lysis plaque phenotypic variation through three passages of infection and harvest of selected plaque forming units (PFU) depending on different plaque aspects such as size, shape, presence or absence of stressed cells on the plaque periphery and interior. Initially, following the first infection with the 2022 MPXV sample, we observed considerable variation in plaque size, although all MPXV lysis plaques were considered small. This was particularly evident when MPXV plaques were compared to VACV-Western Reserve (VACV-WR) plaques, the latter of which were used as a reference point in this study since VACV is well-characterized in the poxvirus literature. After careful PFU evaluation and selection, a total of ten potential clones were collected for the second infection. However, despite selecting ten distinct potential MPXV clones, most of the PFU variation was considerably reduced after the second and third infections rounds, as we had anticipated, consistent with the expected effects of clonal purification, which separates co-existing phenotypes clones. Following an in-depth analysis of all the potential MPXV clones, we successfully obtained three MPXV clones from the 2022 outbreak in Brazil.

**Figure 1:**
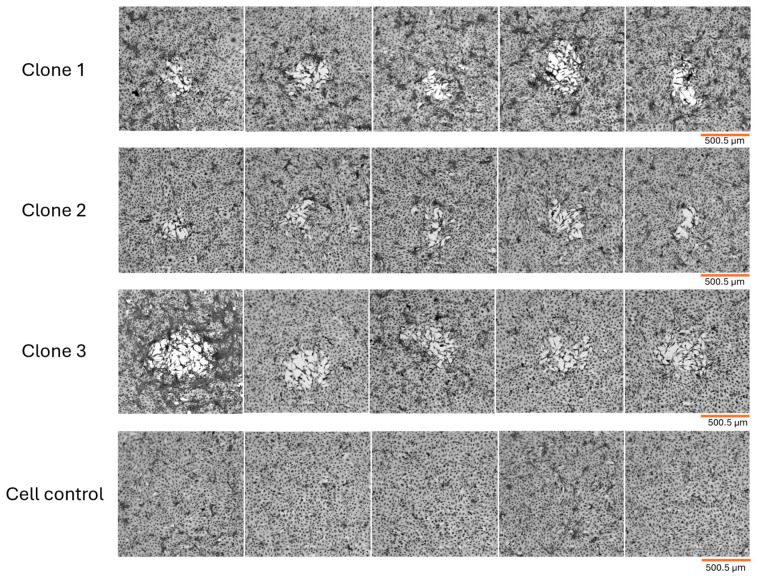
MPXV clone lysis plaques in BSC-40 cell monolayer 48 hpi. Fixed cells were stained with crystal violet and observed under the microscope (SOPTOP PLAN EX30—4×/0.10 magnification)—scale bar in orange: 500.5 µm. Five different plaques can be observed for clones 1, 2 and 3, respectively. Uninfected cell control is also represented. MPXV clone lysis plaques are overall irregular in shape and slightly different in size. For all three clones stressed cells are observed within plaques.

The three MPXV clones obtained exhibited an irregular format, maintaining this characteristic from the early infection. We observed that the plaque size for each clone was roughly the same when comparing plaques from the same clone after 48 h of infection. Precisely measuring lysis plaques was not our primary objective, given the irregular nature of MPXV plaques, but the length of a total of ten plaques per final isolated clone were randomly measured and the average plaque size was calculated. However, when we compared the plaques from the three MPXV clones, we observed slight size variations, with clone 2 presenting the smallest plaques with 273.45 µm average length, followed by clone 1 with 362.34 µm, and clone 3 with 493.72 µm, approximately. All plaques presented stressed cells at the periphery and stressed cells or cell debris within (Figure 1). Moreover, in an attempt to observe more pronounced variation in plaque size among the final isolated clones, we also evaluated the plaque phenotypes after 72 h of infection. However, the longer incubation period did not result in larger lysis plaques forming, so we maintained a 48 h incubation period.

Overall, we observed that the Brazilian MPXV isolate from the 2022 outbreak produced irregularly shaped, small lysis plaques in BSC-40 cells incubated with agar-enriched minimal essential medium (MEM) after 48 h of infection. The Brazilian MPXV isolate exhibited phenotypic variation, enabling the selection and isolation of distinct clones, which were characterized in subsequent sections of this study.

### 3.2. MPXV Clones Comet-Forming Tendencies

**Figure 2:**
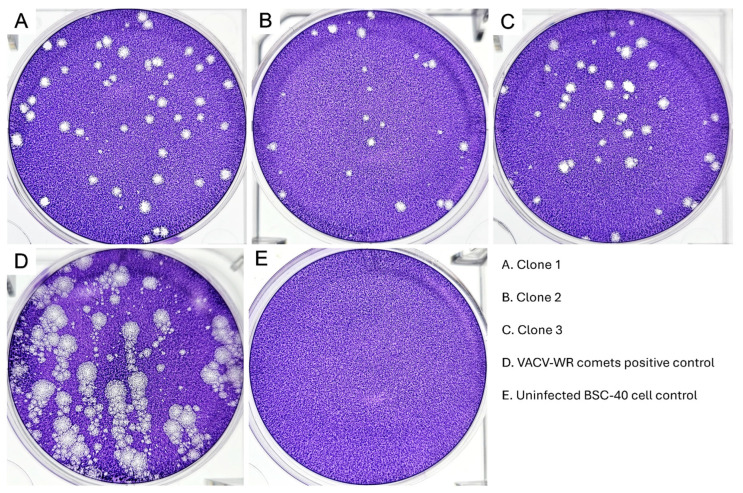
Evaluation of EEV morphotype liberation through comet forming capacity assay. BSC-40 monolayers were fixed at 72 hpi and stained with crystal violet for plaque visualization. Clones 1, 2 and 3 can be observed in (**A**–**C**). VACV-WR was included as a positive control for comet formation (**D**). Uninfected BSC-40 cell control in (**E**). Visualization of plates over a transilluminator. None of the MPXV clones were able to form comets.

To evaluate the Brazilian 2022 outbreak MPXV isolate clone dissemination through the liberation of the extracellular enveloped virus (EEV), we have performed a comet-forming assay. For comparison purposes, we included VACV-WR as a positive control, since this virus is known to release large quantities of EEVs, producing large, well-defined comets [43,44,45,46]. In our analysis of the comet-forming assay, none of the three Brazilian isolated MPXV clones formed comets, in contrast to the VACV-WR positive control (Figure 2). When the fixed and stained BSC-40 monolayers were observed over a transilluminator, we observed all MPXV clones formed small plaques that did not present any projections, as would be expected if the clones had formed comets.

An important aspect of the Brazilian 2022 MPXV outbreak isolate’s inability to form comets is that this same sample had its multiplication cycle analyzed using an ultrastructural approach, which revealed the absence of EEV morphotypes [47]. In our previous work, transmission electron microscopy of MPXV-infected VERO cells enabled us to study the virus multiplication cycle in detail, including the morphogenesis of new particles. While MPXV morphogenesis remained consistent with what is documented in the poxvirus literature, we have mainly observed the formation of the intracellular mature virus (IMV) morphotype [47]. This suggests a probable low production of the intermediate morphotype, the immature enveloped virus (IEV), and consequently, low or no production of EEV.

As we know, the production and liberation of EEV is a notable feature of the multiplication cycle of many poxviruses. EEV liberation is considered one of the most important aspects of poxvirus biology regarding long-distance cell-to-cell dissemination within tissue and evasion of the host immune system, both of which correlate with virus virulence [44,45,46]. Like VACV and other poxviruses, MPXV can produce and release EEV morphotype particles, which induce the formation of comets [45,48]. Distinct MPXV strains of both clades I and II can form comets since this characteristic is well-conserved; however, it is worth noticing that the clade IIb.1 presents markedly reduced comet formation in comparison to other MPXV strains [49]. Furthermore, the observed characteristics of the lysis plaques produced by the 2022 MPXV sample in BSC-40, as well as the absence of comet formation, are similar to those previously described by McGrail et al. (2024) when comparing a 2022 MPXV outbreak sample with the USA 2003 strain and the WRAIR 7-61 strain [50]. Overall, the characterization of the 2022 MPXV lysis plaque phenotype and comet formation tendencies in BSC-40 indicates diminished viral growth and reduced dissemination through EEV liberation, which differs from what can be observed in MPXV clade II strains prior to the clade IIb emergence in 2022.

### 3.3. Genomic Analysis of MPXV Clones

To further explore the differences among the clones of the Brazilian 2022 MPXV outbreak isolate, we sequenced the clones’ genomes and performed an exploratory genomic analysis. The genome assemblies revealed scaffolds of size 197,243 bp for clone 1, 197,209 bp for clone 2, and 197,121 bp for clone 3. Complete sequencing, assembly, and prediction data can be found in Table 1, as well as access codes for the NCBI database, GenBank. During the quality assessment of the assemblies, we observed a high number of degenerate bases in clone 1—accounting for 9.42% of the genome (18,563 bp)—and in clone 3—accounting for 12% of the genome (23,660 bp). The clone 2 genome was found to be the most complete, leading to its interpretation as the closest representation of the actual state of the genome (Table 1). This information is significant because it may have ramifications for further analyses, including coding DNA sequences (CDS) prediction. For the comparative purpose of this study, we opted to utilize all genomes for subsequent analysis.

Following the genome sequencing and assembly, we inferred a phylogeny from MPVX circulating in Brazil during the initial 2022 Clade IIb wave, in order to evaluate inter-clone similarity and place them in context with published sequences. Genomes were aligned (open reading frames (ORFs) OPG001–OPG210) using MAFFT v7.450, then trimmed with ClipKIT v0.1.0, yielding a final alignment of 187,913 nucleotide positions after gap filtering and site trimming. A maximum-likelihood tree was inferred in IQ-TREE 2 with the substitution model General Time Reversible with empirical base frequencies and FreeRate heterogeneity with 5 rate categories (GTR+F+R5), and node support was assessed with 1000 bootstraps.

As expected, the three clones formed a cohesive cluster with within Clade IIb Brazilian B.1 genomes. The clonal sequences formed a single, well-supported clade (Supplementary Figure S1). Notably, clones 1 and 3 were more similar to each other and appeared to harbor more mutations than clone 2, which seemed comparatively conserved relative to other contemporaneous genomes.

**Figure 3:**
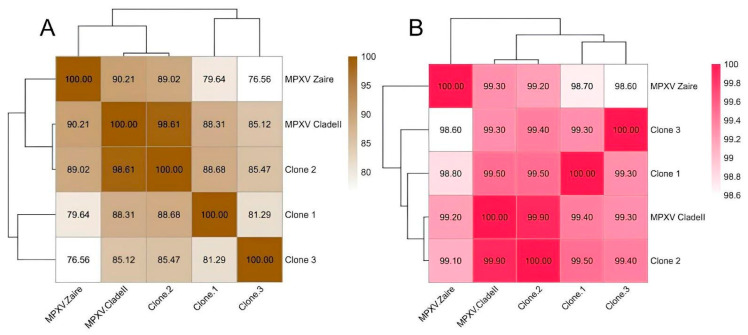
Heatmaps comparing the clone genomes between each other and the references. (**A**) Genomes identities between the clade I reference (MPXV Zaire), clade II reference and the clones generated in this work. (**B**) Average nucleotide identities between the clade I reference (MPXV Zaire), clade II reference and the clones generated in this work.

To facilitate broader understanding and comparison, we included two reference genomes in the next analysis: one for each MPXV clade. The Zaire 1979-005 strain (GenBank accession number HM172544) was used as a reference for clade I, and the MPXV 2022 strain (GenBank accession number NC_063383) for clade II. Figure 3A shows the comparison between each genomic sequence used in this study, highlighting a relative dissimilarity among them when comparing with the references. As cited before, clones 1 and 3 had a high number of Ns in the sequence, due to the quality of the sequencing, which explains this variation. Even so, it is observed that this variation increases the more phylogenetically distant the comparison pair is, showing that, even with unknown bases, the depth of sequencing generated data for a good assembly. The genome of clone 2, considered the best sequenced, has a high similarity to that of the clade II reference, but we observed a variation of about 2% of the genome, which will be evaluated below.

Additionally, we evaluated the percentage of shared sequence identity between the complete genomes of the MPXV clones and the references (Figure 3B). This result is more consistent with expectations, with genes in clade II being more similar to each other. This is because the analysis only uses homologous sequences present in all genomes, which minimizes the impact of Ns. In general, all genomes exhibit high levels of shared identity, with a minimum of 98.6% between clone 3 and the Zaire strain. The three clones share nearly 99.5% similarity with each other, while interestingly, clone 2 showed the highest percentage of shared identity (99.9%) with the MPXV 2022 reference strain, followed by clones 1 and 3 with 99.4% and 99.3%, respectively.

ORFs were predicted using Prodigal and annotated using blastn, HHpred and InterproScan. The annotation showed that clone 1 contained 209 predicted genes, clone 2 coded for 213 genes, and clone 3 had 194 genes. For comparison, the MPXV Zaire strain has 197 coding sequences, while MPXV clade II has 179. Despite the variation in the number of genes among the clones, which could potentially indicate an artifact of genomic assembly and annotation bias, a more thorough examination of the sequences revealed that the composition does not exhibit significant divergence. As we lacked the necessary transcriptome data to validate or reject the predicted sequences, we elected to utilize them as they were provided by the program.

**Figure 4:**
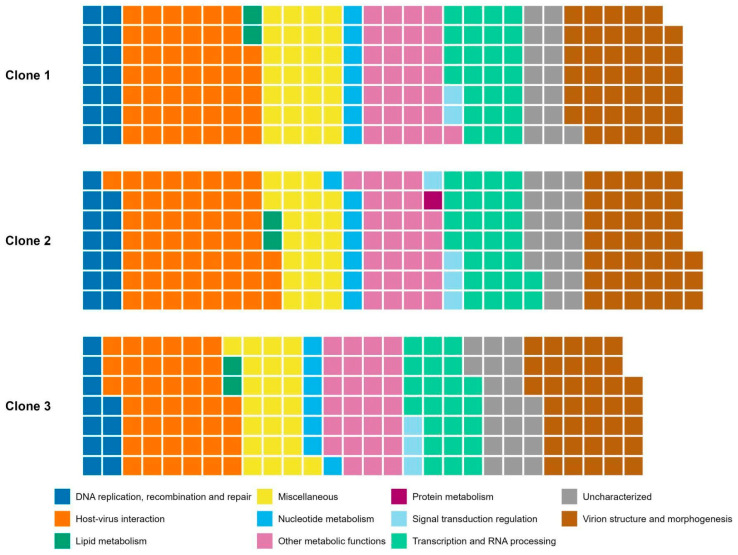
Nucleo-Cytoplasmic Virus Orthologous Groups (NCVOG) classification of MPXV clones’ genes in the format of a waffle plot. Each square is associated with one annotated CDS. Colors indicate different categories of NCVOG classification.

To further evaluate the MPXV clones’ gene composition, we categorized the annotated genes according to the Nucleo-Cytoplasmic Virus Orthologous Groups (NCVOG) classification [51]. Most of the annotated CDS belonged to the category of “Host–virus interaction,” such as cytokine antagonists and entry/fusion complex proteins, followed by “Virion structure and morphogenesis,” such as membrane proteins associated with different infectious forms and DNA packing ATPase A32-like, and “Other metabolic functions”, including proteins with roles that are not yet defined or with more than one role, such as telomere-binding proteins and glutaredoxins, for example. NCVOG categories with the least annotated CDSs were ‘Signal transduction regulation’, ‘Lipid metabolism’—lipases—and ‘Protein metabolism’, the latter being a metalloendopeptidase found only in clone 2 (Figure 4).

**Figure 5:**
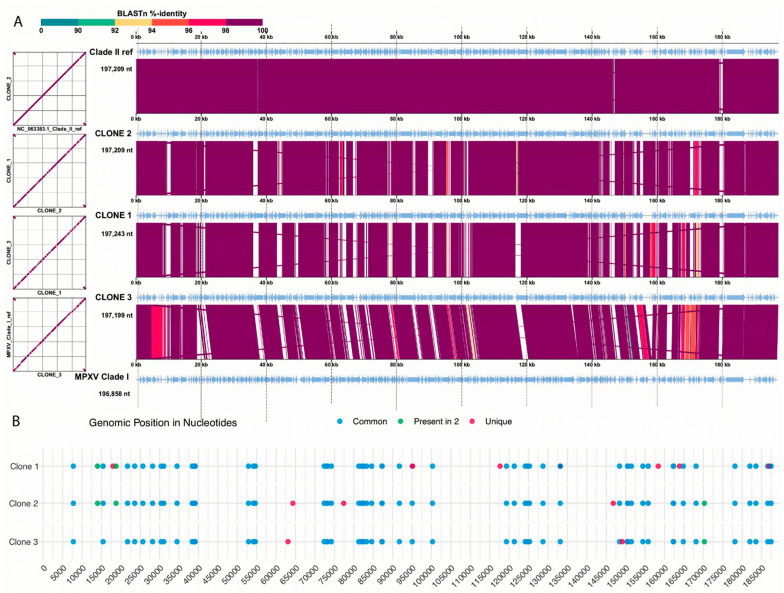
Nucleotide sequence comparison of the genomes. (**A**) Synteny analysis between the genomes. The white spaces can be tracked as the regions of unknown bases. (**B**) Analysis of single nucleotide variant of each clone genome against the Clade II reference. Blue dots—common SNVs; green—SNVs present in two genomes; red dots—SNVs unique for each genome.

Synteny analysis confirmed the similarity results, with clones 2 and 3 presenting a distinct dissimilarity close to the 14 kb mark of the genome. Single nucleotide variation (SNV) analysis showed a similar result with most of the mutations present in proximity to the 14 kb mark and close to the last portion of the pox genome. Using an in-house script (https://github.com/iza-mcac/2025-10-Pox-Variant_calling (accessed on 12 September 2025)) we called SNVs in all the ORFs associated with the annotated CDS, which found from 54 to 60 SNVs present in each clone (Figure 5). Among these SNVs we found two variants in common between two clones and ten variants that were clone specific (Figure 5B).

To facilitate understanding, the SNVs have been condensed into a select group that exclusively comprises those located within a standard pox ORF, as detailed in Table 2. A subsequent analysis of these mutations reveals a preponderance of synonymous mutations (14/18), followed by non-synonymous mutations (4/18). Among the genes exhibiting the most mutations, DNA-dependent RNA polymerase is the main variation hotspot with six SNVs, followed by the B22R family serpin with three SNVs. The C4L/C10L-like family protein, OPG037-homolog ankyrin repeat domain containing-protein and schlafen had two SNVs each, while EGF-like domain containing-protein, OPG025-homolog ankyrin repeat domain containing-protein and DNA helicase had only one. As indicated by Yadav et al. (2023), the functions of the mutated genes are associated with DNA replication, transcription, and interaction with the host [52]. Considering these associated functions to genes and identified mutations, we have hypothesized that some of the found SNVs could possibly be correlated to the phenotypic plaque variation observed among MPXV clones to some degree.

To support that the SNVs found on MPXV clones are representative of the intra-clonal diversity of the MPXV original sample, we also performed SNV calling on the raw sequencing data of the original sample by Guimarães et al. (2023) and found that 16 of the 18 mutations in Table 2 were present in the original sample [14]. This emphasizes that these mutations did not arise from the experimental passages. Specifically, the C>T mutation at position 83,326 and the G>A mutation at position 124,130—the non-synonymous ones—were observed with counts above 900 in the reads of the original sample). These data show that the expected bottleneck effect of the passages was not a relevant selective factor for the SNVs found.

**Table 2:** Mutation found on MPXV clones CDSs through SNV calling compared to clade II reference. From left to right columns: position of mutation, nucleotide within reference genome, altered nucleotide description of CDS, variant type (non-synonymous (NSY), synonymous (SY) or non-applicable (NA) for the original sample genome and for clones 1, 2, and 3.

MPXV ORF ^a^	Position	Ref	Alt	Description	Original Sample ^b^	Clone 1	Clone 2	Clone 3
OPG019	7771	C	T	EGF-like domain containing-protein	SY	SY	SY	SY
OPG025	14000	G	T	Ankyrin repeat domain containing-protein	SY	SY	SY	NA
OPG031	17961	A	G	C4L/C10L-like family protein	NA	SY	SY	SY
OPG031	18769	A	G	C4L/C10L-like family protein	SY	SY	SY	NA
OPG037	21723	G	A	Ankyrin repeat domain containing-protein	SY	SY	SY	SY
OPG037	23564	C	T	Ankyrin repeat domain containing-protein	SY	SY	SY	SY
OPG105	81275	G	A	DNA-dependent RNA polymerase	SY	SY	SY	SY
OPG105	81977	A	G	DNA-dependent RNA polymerase	SY	SY	SY	SY
OPG105	82373	C	T	DNA-dependent RNA polymerase	SY	SY	SY	SY
OPG105	82451	G	A	DNA-dependent RNA polymerase	SY	SY	SY	SY
OPG105	83326	C	T	DNA-dependent RNA polymerase	NSY	NSY	NSY	NSY
OPG105	84587	C	T	DNA-dependent RNA polymerase	SY	SY	SY	SY
OPG145	124130	G	A	DNA helicase	NSY	NSY	NSY	NSY
OPG188	162243	G	A	Schlafen	SY	SY	SY	SY
OPG188	162331	C	T	Schlafen	SY	SY	SY	SY
OPG210	183519	C	T	B22R family serpin	NSY	NSY	NSY	NSY
OPG210	186578	G	A	B22R family serpin	NSY	NSY	NSY	NSY
OPG210	186933	A	T	B22R family serpin	NA	SY	SY	SY

^a^ Orthopoxviruses canonical ORF nomenclature. ^b^ Sequencing from the original sample [14].

Utilizing these data, a three-gene subset with the non-synonymous mutations was isolated and subjected to further investigation to ascertain whether these mutations would impact the biology of the clone, employing structural modeling. The first ORF selected was the catalytic domain of DNA-dependent RNA polymerase, which presented a variation in a cytosine in reference to a thymine in the clones in position 83,326, resulting in the substitution of a serine residue, a nonpolar amino acid, to leucine, a polar amino acid. This change had a score of −1 in the BLOSUM62 matrix. To build the tertiary structures, SwissModel presented a good model based on high-quality templates, which led us to use the server service for modeling (Table 3).

The mapping process revealed the location of the variation within the bridge helix (BH) of the cleft (Supplementary Figure S2). This structure, consisting of an alpha helix, functions as a wall that divides the nucleic acid loading channels into a primary channel, which accommodates double-stranded DNA to be transcribed, and a secondary channel, into which free NTPs enter and are accommodated for base pairing [53]. Furthermore, evidence suggests that BH plays a role in a transcription checkpoint function, as demonstrated by the ability of a threonine residue to serve as a probe, indicating the stability of the nascent RNA molecule. This process can reveal potential pairing errors, which in turn can result in backtracking [54]. Our in silico analyses did not indicate major changes in the structure of the clone molecules, with no new atomic interactions or variation in the volume of the mutation site (Supplementary Figure S3). The predicted ΔΔG for this mutation was calculated as −0.109 Kcal, indicating that this variant stabilizes the structure. Furthermore, a thorough review of the existing literature revealed the absence of any functions, other than structural ones, for the initial serine. However, we cannot rule out the possibility that this mutation may impact the function of the molecule. RNA polymerase is an enzyme that plays an essential role in the cycle of these viruses, so mutations so close to relevant sites that alter the polarity of the region may generate functional changes, albeit not very significant ones. As observed, these clones appear to generate smaller plaques, so it could be inferred that this mutation has a slight influence on this characteristic. Nevertheless, further investigation is necessary to ascertain the extent to which this mutation impacts the MPXV clone multiplication cycle, if any.

The subsequent ORF was related to DNA helicase, wherein a guanine in the reference genome was substituted with an adenine in the clones, resulting in the replacement of glutamate, an acidic amino acid, with lysine, a basic amino acid. This substitution has a score of 1 in the BLOSUM62 matrix. As template-dependent methods did not generate good models, we used AlphaFold2, via ColabFold, to infer the structures (Table 4). DNA helicase has been hypothesized to play a role in RNA transcription termination and DNA repair, though this remains to be substantiated [55,56]. Considering the mutation found here, it was established that this variation was not located in the functional domain of the molecule (Supplementary Figure S4). Consequently, it was not possible to ascertain whether there were functional changes. Ramachandran plots (Supplementary Figure S5) did not show major differences, as well as ProtParam indexes. In addition, electrostatics analyses performed in PyMol also did not present changes, showing that sites of putative interaction maintain similar. However, the position was in close proximity to the 5′ end of this domain, not only in secondary structure (61 amino acids distant) but also spatially (14.8 Å) and has a predicted ΔΔG calculated as 0.775 kcal, indicating that this variant destabilizes the structure. Therefore, it is plausible to hypothesize that these mutant molecules may exhibit deficiencies in activity in comparison to the naive molecule, as evidenced by their thermodynamic variations.

Finally, the ORF related to the B22R family serpin had two single nucleotide variations, one from cytosine to thymine and the other from guanine to adenine. The first substitution resulted in the replacement of proline with serine in position 722, both of which are uncharged polar. The second substitution involved the replacement of methionine with isoleucine, both of which are also nonpolar. The scores for both substitutions are 4 in the BLOSUM62 matrix. B22 proteins have been demonstrated to play a role in the inactivation of host T cells, a process that is crucial for virulence. Consequently, mutants lacking this gene have been shown to exhibit an attenuated infection [57]. However, attempts to reconstruct the molecule using computational methods have generated models with low reliability, which has prevented the utilization of any model for analyses involving catalytic sites and interaction pockets.

The integration of these results yielded no compelling evidence to suggest a potential phenotypic consequence, such the ones cited before. The mutation in RNA polymerase appears to cause a molecular imbalance; however, further analysis is required to indicate causality. Mutations in B22R could not be analyzed at the structural level because the models generated were not reliable. As demonstrated by Delamonica et al. (2023), the APOBEC-3 enzyme, which is part of the innate immune system against viruses, causes C > T and G > A mutations, thereby exerting pressure on the emergence of these mutations in MPXV [58]. It was demonstrated that epitopes against the OPG105 and OPG220 genes were identified in various orthopoxvirus clades, indicating them as possible targets for immune response [59,60]. In this sense, it is not possible to conclude that the 2022 outbreak was primarily driven by vaccine escape from mutations in these two genes. However, our data underscores the prevalence of this variability, thereby classifying these regions as mutation hotspots.

## 4. Conclusions

Herein, we have evaluated the biological diversity of an MPXV sample from 2022 early outbreak through phenotypic and genomic characterization. The three distinct selected MPXV clones exhibited lysis plaques of irregular shape and reduced size in BSC-40 after 48 h of incubation. Compared to the VACV-WR strain, the MPXV clones did not produce comets when evaluated for EEV liberation, indicating a low rate of EEV liberation. Regarding the phenotypic characterization of MPXV lysis plaques, our results suggest a low level of viral spread in vitro, which is consistent with recent descriptions of the 2022 MPXV IIb strain in the literature. Nevertheless, the usage of alternative cell lineages may present different results for in vitro phenotypic characterization of MPXV.

Regarding the genomic characteristics of the clones, we have found that the genes did not deviate in function from the reference. However, additional sequences were identified in the strains under investigation. Subsequent analysis of the composition of each gene, in addition to a review of the extant literature, demonstrated that the majority of these extra sequences are, in fact, genes that have lost their function, generating truncated transcripts [61]. These sequences were found in variable regions and were associated with functions related to virus–host interaction.

Nevertheless, a high level of SNVs was identified, which is analogous to that observed when comparing different MPXV lineages from various global regions during the 2022 outbreak [62]. In addition to our data, the frequency of allele variation in the raw reads of the original sample supports the idea that clonal SNVs are well represented within the sample, thus demonstrating allelic heterogeneity within a single MPXV-derived lesion. These variations were identified in genes that have previously been documented as being highly variable, as they are known to be targeted by APOBEC-3. Our findings are consistent with those reported by Vauhkonen et al. (2023), which indicate that substantial variations in MPXV occur within the host, with samples from the same lesion exhibiting viral clones that exhibit significant variations [63]. The findings of both studies underscore the correlation between these mutations and the activity of APOBEC-3, as the enzyme’s distinctive signature has been identified.

Altogether, the clonal phenotypic and genomic characterization of the Brazilian 2022 MPXV isolate has provided data that supports the current knowledge of MPXV through yet a different approach. Our findings suggest that although conserved, it is possible that distinct MPXV 2022 IIb variants may circulate together and can be found within a single exanthematous lesion. However, to further demonstrate the extent to which subpopulation variants are present and co-circulating after the 2022 MPXV emergence, replicates of these analyses should be done to a more representative dataset of lesion samples. Finally, the results hereby presented contribute to our understanding of the virus’s biology, corroborating previous findings while offering a new perspective on the viral characterization.

## Figures and Tables

**Table 1:** General genomic features of MPXV clones generated in this study.

Clone	Sequencing Coverage	Assembly Size (bp)	CDS #	GC Content (%)	GenBank Access
Clone 1	447x	197,243	209	34.72	PX448464
Clone 2	591x	197,209	213	33.15	PX448465
Clone 3	760x	197,121	194	35.3	PX448466

**Table 3:** Information about the tertiary structure models of the DNA-dependent RNA-polymerase used in this work, performed in the SwissModel server.

Isolate	Template	Organism	Name	Parameters		
				GMQE	Identity	Coverage
Clade II	8p0k.1.A	Vaccinia virus	DNA-directed RNA polymerase 147 kDa polypeptide	0.86	99.1	1
Clone 1	6rid.1	Vaccinia virus	DNA-dependent RNA polymerase subunit rpo147	0.85	98.99	1
Clone 2	8p0k.1.A	Vaccinia virus	DNA-directed RNA polymerase 147 kDa polypeptide	0.87	99	1
Clone 3	6rid.1	Vaccinia virus	DNA-dependent RNA polymerase subunit rpo147	0.85	98.99	1

**Table 4:** Information about the tertiary structure models of the DNA helicase used in this work, performed in the ColabFold server.

Isolate	Homolog ^a^	Organism	Name	Parameters		
				Prob ^b^	SeqId ^c^	TM-Score
Clade II	A0A2L1GHX6	Saltwater crocodilepox virus	DNA helicase	1	39.4	0.933
Clone 1	A0A2L1GHX6	Saltwater crocodilepox virus	DNA helicase	1	39.8	0.932
Clone 2	A0A2L1GHX6	Saltwater crocodilepox virus	DNA helicase	1	39.4	0.927
Clone 3	A0A2L1GHX6	Saltwater crocodilepox virus	DNA helicase	1	39.5	0.932

^a^ BFVD code. ^b^ Probability. ^c^ Sequence Identity.

## Data Availability

The raw sequencing data used in this work are available at the NCBI SRA under accession PRJNA1345768. FASTA files for the three genomes generated and the respective gene annotations are available at the NCBI Genbank under accessions PX448464 (clone 1), PX448465 (clone 2) and PX448466 (clone 3).

## Supplementary Materials

The following supporting information can be downloaded at: https://www.mdpi.com/article/10.3390/v18010062/s1, Figure S1:Phylogeny of Brazilian MPXV genomes contemporaneous with the study clones (2022); Figure S2: Cartoon representation of the position of the mutation in the DNA-dependent RNA polymerase gene; Figure S3: Ramachandran plots of Clade II, Clone 1, Clone 2 and Clone 3 DNA-dependent RNA polymerase models; Figure S4: Cartoon representation of the position of the mutation in the DNA helicase gene; Figure S5: Ramachandran plots of Clade II, Clone 1, Clone 2 and Clone 3 DNA helicase models.

## References

[1] von Magnus P., Andersen E.K., Petersen K.B., Birch-Andersen A. (1959). A pox-like disease in Cynomolgus monkeys. Acta Pathol. Microbiol. Scand..

[2] Marennikova S.S., 9eluhina T.M., Mal’ceva N.N., Cmi1kjan K.L., Macevit G.R. (1972). Isolation and Properties of the Causal Agent of a New Variola-like Disease (Monkeypox) in Man. Bull. World Health Organ..

[3] World Health Organization (WHO) Global Mpox (Monkeypox) Situation Dashboard. https://worldhealthorg.shinyapps.io/mpx_global/.

[4] Happi C., Adetifa I., Mbala P., Njouom R., Nakoune E., Happi A., Ndodo N., Ayansola O., Mboowa G., Bedford T. (2022). Urgent Need for a Non-Discriminatory and Non-Stigmatizing Nomenclature for Monkeypox Virus. PLoS Biol..

[5] Bunge E.M., Hoet B., Chen L., Lienert F., Weidenthaler H., Baer L.R., Steffen R. (2022). The Changing Epidemiology of Human Monkeypox—A Potential Threat? A Systematic Review. PLoS Negl. Trop. Dis..

[6] Djuicy D.D., Sadeuh-Mba S.A., Bilounga C.N., Yonga M.G., Tchatchueng-Mbougua J.B., Essima G.D., Esso L., Nguidjol I.M.E., Metomb S.F., Chebo C. (2024). Concurrent Clade I and Clade II Monkeypox Virus Circulation, Cameroon, 1979–2022. Emerg. Infect. Dis..

[7] Desingu P.A., Rubeni T.P., Sundaresan N.R. (2022). Evolution of Monkeypox Virus from 2017 to 2022: In the Light of Point Mutations. Front. Microbiol..

[8] Luna N., Muñoz M., Bonilla-Aldana D.K., Patiño L.H., Kasminskaya Y., Paniz-Mondolfi A., Ramírez J.D. (2023). Monkeypox Virus (MPXV) Genomics: A Mutational and Phylogenomic Analyses of B.1 Lineages. Travel. Med. Infect. Dis..

[9] Alakunle E., Kolawole D., Diaz-Cánova D., Alele F., Adegboye O., Moens U., Okeke M.I. (2024). A Comprehensive Review of Monkeypox Virus and Mpox Characteristics. Front. Cell Infect. Microbiol..

[10] Mauldin M.R., McCollum A.M., Nakazawa Y.J., Mandra A., Whitehouse E.R., Davidson W., Zhao H., Gao J., Li Y., Doty J. (2022). Exportation of Monkeypox Virus From the African Continent. J. Infect. Dis..

[11] Yinka-Ogunleye A., Aruna O., Dalhat M., Ogoina D., McCollum A., Disu Y., Mamadu I., Akinpelu A., Ahmad A., Burga J. (2019). Outbreak of Human Monkeypox in Nigeria in 2017–18: A Clinical and Epidemiological Report. Lancet Infect Dis.

[12] Oliveira G., Assis F., Almeida G., Albarnaz J., Lima M., Andrade A.C., Calixto R., Oliveira C., Neto J.D., Trindade G. (2015). From Lesions to Viral Clones: Biological and Molecular Diversity amongst Autochthonous Brazilian Vaccinia Virus. Viruses.

[13] Brennan G., Stoian A.M.M., Yu H., Rahman M.J., Banerjee S., Stroup J.N., Park C., Tazi L., Rothenburg S. (2023). Molecular Mechanisms of Poxvirus Evolution. mBio.

[14] Guimarães N.R., Tomé L.M.R., Lamounier L.O., Silva M.V.F., Lima M.T., da Costa A.V.B., Luiz K.C.M., de Jesus R., Trindade G.d.S., Oliveira D.B. (2023). Genomic Surveillance of Monkeypox Virus, Minas Gerais, Brazil, 2022. Emerg. Infect. Dis..

[15] Li Y., Olson V.A., Laue T., Laker M.T., Damon I.K. (2006). Detection of Monkeypox Virus with Real-Time PCR Assays. J. Clin. Virol..

[16] Bolger A.M., Lohse M., Usadel B. (2014). Trimmomatic: A Flexible Trimmer for Illumina Sequence Data. Bioinformatics.

[17] Li H. (2018). Minimap2: Pairwise Alignment for Nucleotide Sequences. Bioinformatics.

[18] Abueg L.A.L., Afgan E., Allart O., Awan A.H., Bacon W.A., Baker D., Bassetti M., Batut B., Bernt M., Blankenberg D. (2024). The Galaxy Platform for Accessible, Reproducible, and Collaborative Data Analyses: 2024 Update. Nucleic Acids Res..

[19] Bankevich A., Nurk S., Antipov D., Gurevich A.A., Dvorkin M., Kulikov A.S., Lesin V.M., Nikolenko S.I., Pham S., Prjibelski A.D. (2012). SPAdes: A New Genome Assembly Algorithm and Its Applications to Single-Cell Sequencing. J. Comput. Biol..

[20] Kearse M., Moir R., Wilson A., Stones-Havas S., Cheung M., Sturrock S., Buxton S., Cooper A., Markowitz S., Duran C. (2012). Geneious Basic: An Integrated and Extendable Desktop Software Platform for the Organization and Analysis of Sequence Data. Bioinformatics.

[21] Katoh K., Standley D.M. (2013). MAFFT Multiple Sequence Alignment Software Version 7: Improvements in Performance and Usability. Mol. Biol. Evol..

[22] Hyatt D., Chen G.-L., Locascio P.F., Land M.L., Larimer F.W., Hauser L.J. (2010). Prodigal: Prokaryotic Gene Recognition and Translation Initiation Site Identification. BMC Bioinform..

[23] Carvalho J.V.R.P., Carlson R.M., Ghosh J., Queiroz V.F., de Oliveira E.G., Botelho B.B., Filho C.A.C., Agarkova I.V., McClung O.W., Van Etten J.L. (2024). Genomics and Evolutionary Analysis of Chlorella Variabilis- Infecting Viruses Demarcate Criteria for Defining Species of Giant Viruses. J. Virol..

[24] Altschup S.F., Gish W., Miller W., Myers E.W., Lipman D.J. (1990). Basic Local Alignment Search Tool. J. Mol. Biol..

[25] Zimmermann L., Stephens A., Nam S.Z., Rau D., Kübler J., Lozajic M., Gabler F., Söding J., Lupas A.N., Alva V. (2018). A Completely Reimplemented MPI Bioinformatics Toolkit with a New HHpred Server at Its Core. J. Mol. Biol..

[26] Gabler F., Nam S.Z., Till S., Mirdita M., Steinegger M., Söding J., Lupas A.N., Alva V. (2020). Protein Sequence Analysis Using the MPI Bioinformatics Toolkit. Curr. Protoc. Bioinform..

[27] Paysan-Lafosse T., Blum M., Chuguransky S., Grego T., Pinto B.L., Salazar G.A., Bileschi M.L., Bork P., Bridge A., Colwell L. (2023). InterPro in 2022. Nucleic Acids Res.

[28] Jain C., Rodriguez-R L.M., Phillippy A.M., Konstantinidis K.T., Aluru S. (2018). High Throughput ANI Analysis of 90K Prokaryotic Genomes Reveals Clear Species Boundaries. Nat. Commun..

[29] Kolde R. (2025). pheatmap: Pretty Heatmaps. R Package Version 1.0.13. https://github.com/raivokolde/pheatmap.

[30] Nishimura Y., Yamada K., Okazaki Y., Ogata H. (2024). DiGAlign: Versatile and Interactive Visualization of Sequence Alignment for Comparative Genomics. Microbes Env..

[31] Iza M.C.A.C. (2025). In-House Script for Poxvirus SNV Variant Calling. https://github.com/iza-mcac/2025-10-Pox-Variant_calling.

[32] Elbe S., Buckland-Merrett G. (2017). Data, Disease and Diplomacy: GISAID’s Innovative Contribution to Global Health. Glob. Chall..

[33] Steenwyk J.L., Buida T.J., Li Y., Shen X.X., Rokas A. (2020). ClipKIT: A Multiple Sequence Alignment Trimming Software for Accurate Phylogenomic Inference. PLoS Biol..

[34] Minh B.Q., Schmidt H.A., Chernomor O., Schrempf D., Woodhams M.D., Von Haeseler A., Lanfear R., Teeling E. (2020). IQ-TREE 2: New Models and Efficient Methods for Phylogenetic Inference in the Genomic Era. Mol. Biol. Evol..

[35] Kalyaanamoorthy S., Minh B.Q., Wong T.K.F., Von Haeseler A., Jermiin L.S. (2017). ModelFinder: Fast Model Selection for Accurate Phylogenetic Estimates. Nat. Methods.

[36] Letunic I., Bork P. (2024). Interactive Tree of Life (ITOL) v6: Recent Updates to the Phylogenetic Tree Display and Annotation Tool. Nucleic Acids Res..

[37] Waterhouse A., Bertoni M., Bienert S., Studer G., Tauriello G., Gumienny R., Heer F.T., De Beer T.A.P., Rempfer C., Bordoli L. (2018). SWISS-MODEL: Homology Modelling of Protein Structures and Complexes. Nucleic Acids Res..

[38] Mirdita M., Schütze K., Moriwaki Y., Heo L., Ovchinnikov S., Steinegger M. (2022). ColabFold: Making Protein Folding Accessible to All. Nat. Methods.

[39] van Kempen M., Kim S.S., Tumescheit C., Mirdita M., Lee J., Gilchrist C.L.M., Söding J., Steinegger M. (2024). Fast and Accurate Protein Structure Search with Foldseek. Nat. Biotechnol..

[40] Potter S.C., Luciani A., Eddy S.R., Park Y., Lopez R., Finn R.D. (2018). HMMER Web Server: 2018 Update. Nucleic Acids Res..

[41] Laimer J., Hiebl-Flach J., Lengauer D., Lackner P. (2016). MAESTROweb: A Web Server for Structure-Based Protein Stability Prediction. Bioinformatics.

[42] Walker J.M., Gasteiger E., Hoogland C., Gattiker A., Duvaud S., Wilkins M.R., Appel R.D., Bairoch A. (2005). Protein Analysis Tools on the ExPASy Server 571 571 From: The Proteomics Protocols Handbook Edited Protein Identification and Analysis Tools on the ExPASy Server. The Proteomics Protocols Handbook.

[43] Payne L.G. (1980). Significance of Extracellular Enveloped Virus in the in Vitro and in Vivo Dissemination of Vaccinia. J. Gen. Virol..

[44] Smith G.L., Vanderplasschen A., Law M. (2002). Printed in Great Britain The Formation and Function of Extracellular Enveloped Vaccinia Virus. J. Gen. Virol..

[45] Reeves P.M., Smith S.K., Olson V.A., Thorne S.H., Bornmann W., Damon I.K., Kalman D. (2011). Variola and Monkeypox Viruses Utilize Conserved Mechanisms of Virion Motility and Release That Depend on Abl and Src Family Tyrosine Kinases. J. Virol..

[46] Blasco R., Moss B. (1992). Role of Cell-Associated Enveloped Vaccinia Virus in Cell-to-Cell Spread. J. Virol..

[47] Witt A.S.A., Trindade G.d.S., de Souza F.G., Serafim M.S.M., da Costa A.V.B., Silva M.V.F., de Melo Iani F.C., Rodrigues R.A.L., Kroon E.G., Abrahão J.S. (2023). Ultrastructural Analysis of Monkeypox Virus Replication in Vero Cells. J. Med. Virol..

[48] Mucker E.M., Shamblin J.D., Goff A.J., Bell T.M., Reed C., Twenhafel N.A., Chapman J., Mattix M., Alves D., Garry R.F. (2022). Evaluation of Virulence in Cynomolgus Macaques Using a Virus Preparation Enriched for the Extracellular Form of Monkeypox Virus. Viruses.

[49] Americo J.L., Earl P.L., Moss B. (2023). Virulence Differences of Mpox (Monkeypox) Virus Clades I, IIa, and IIb.1 in a Small Animal Model. Proc. Natl. Acad. Sci. USA.

[50] McGrail J.P., Mondolfi A.P., Ramírez J.D., Vidal S., García-Sastre A., Palacios G., Sanchez-Seco M.P., Guerra S. (2024). Comparative Analysis of 2022 Outbreak MPXV and Previous Clade II MPXV. J. Med. Virol..

[51] Yutin N., Wolf Y.I., Raoult D., Koonin E.V. (2009). Eukaryotic Large Nucleo-Cytoplasmic DNA Viruses: Clusters of Orthologous Genes and Reconstruction of Viral Genome Evolution. Virol. J..

[52] Yadav P., Devasurmutt Y., Tatu U. (2023). Phylogenomic and Structural Analysis of the Monkeypox Virus Shows Evolution towards Increased Stability. Viruses.

[53] Zhang G., Campbell E.A., Minakhin L., Richter C., Severinov K., Darst S.A. (1999). Crystal Structure of Thermus Aquaticus Core RNA Polymerase at 3.3 A ˚ Resolution. Cell.

[54] Da L.T., Pardo-Avila F., Xu L., Silva D.A., Zhang L., Gao X., Wang D., Huang X. (2016). Bridge Helix Bending Promotes RNA Polymerase II Backtracking through a Critical and Conserved Threonine Residue. Nat. Commun..

[55] Simpson D.A., Condit R.C. (1995). Vaccinia Virus Gene A18R Encodes an Essential DNA Helicase. J. Virol..

[56] Senkevich T.G., Katsafanas G.C., Weisberg A., Olano L.R., Moss B. (2017). Identification of Vaccinia Virus Replisome and Transcriptome Proteins by Isolation of Proteins on Nascent DNA Coupled with Mass Spectrometry. J. Virol..

[57] Alzhanova D., Hammarlund E., Reed J., Meermeier E., Rawlings S., Ray C.A., Edwards D.M., Bimber B., Legasse A., Planer S. (2014). T Cell Inactivation by Poxviral B22 Family Proteins Increases Viral Virulence. PLoS Pathog..

[58] Delamonica B., Davalos L., Larijani M., Anthony S.J., Liu J., MacCarthy T. (2023). Evolutionary Potential of the Monkeypox Genome Arising from Interactions with Human APOBEC3 Enzymes. Virus Evol..

[59] Hammarlund E., Lewis M.W., Carter S.V., Amanna I., Hansen S.G., Strelow L.I., Wong S.W., Yoshihara P., Hanifin J.M., Slifka M.K. (2005). Multiple Diagnostic Techniques Identify Previously Vaccinated Individuals with Protective Immunity against Monkeypox. Nat. Med..

[60] Terajima M., Cruz J., Leporati A.M., Demkowicz W.E., Kennedy J.S., Ennis F.A. (2006). Identification of Vaccinia CD8+ T-Cell Epitopes Conserved among Vaccinia and Variola Viruses Restricted by Common MHC Class I Molecules, HLA-A2 or HLA-B7. Hum. Immunol..

[61] Young B., Seifert S.N., Lawson C., Koehler H. (2024). Exploring the Genomic Basis of Mpox Virus-Host Transmission and Pathogenesis. mSphere.

[62] Wang L., Shang J., Weng S., Aliyari S.R., Ji C., Cheng G., Wu A. (2023). Genomic Annotation and Molecular Evolution of Monkeypox Virus Outbreak in 2022. J. Med. Virol..

[63] Vauhkonen H., Kallio-Kokko H., Hiltunen-Back E., Lönnqvist L., Leppäaho-Lakka J., Mannonen L., Kant R., Sironen T., Kurkela S., Lappalainen M. (2023). Intrahost Monkeypox Virus Genome Variation in Patient with Early Infection, Finland, 2022. Emerg. Infect. Dis..

