# Supplementary material for: Unveiling Intra-Clonal Diversity of Monkeypox Virus from Brazil’s First Outbreak Wave
Source: Viruses. 2025 Dec 31;18(1):62. doi: 10.3390/v18010062 (PMC12846513; doi:10.3390/v18010062)
Supplement: Supplementary file 1 [file viruses-18-00062-s001.zip › viruses-3991123-supplementary.pdf]

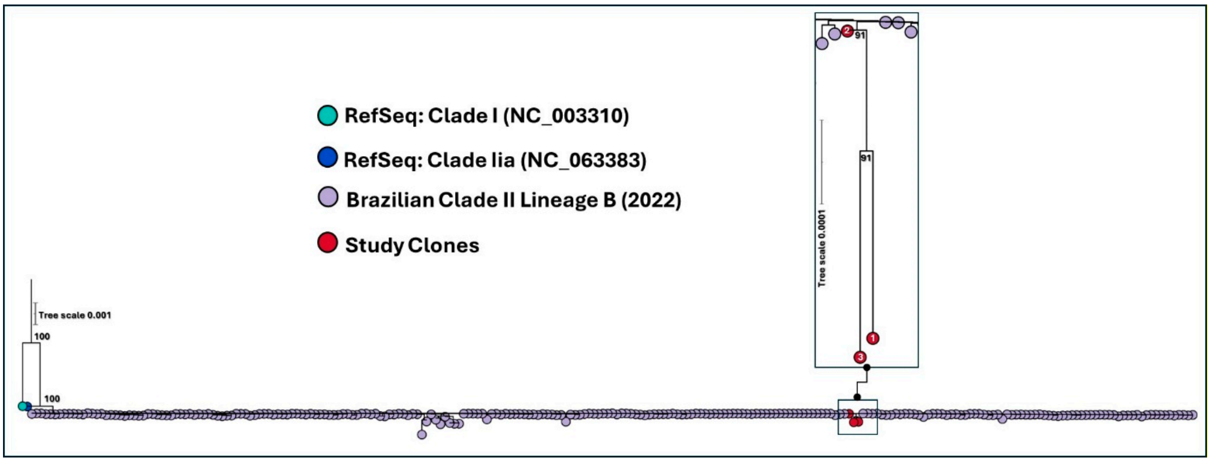

Supplementary Figure S1. Phylogeny of Brazilian MPV genomes contemporaneous with the study clones (2022). Maximum-likelihood tree inferred from 248 Brazilian genomes plus the three study clones, alongside MPV RefSeqs for Clade I (NC_003310) and Clade IIa (NC_063383), aligned over 187,913 nucleotide sites. The best-fit substitution model was GTR+F+R5 with bootstrap support based on 1,000 replicates. Colored circles denote categories: teal = Clade I RefSeq (NC_003310); dark blue = Clade IIa RefSeq (NC_063383); light purple = Brazilian Clade II, Lineage B (2022); red = study clones. The clones were identified by their corresponding numbers in white. The scale bar indicates substitutions per site. The inset highlights the clonal cluster (bootstrap = 91.

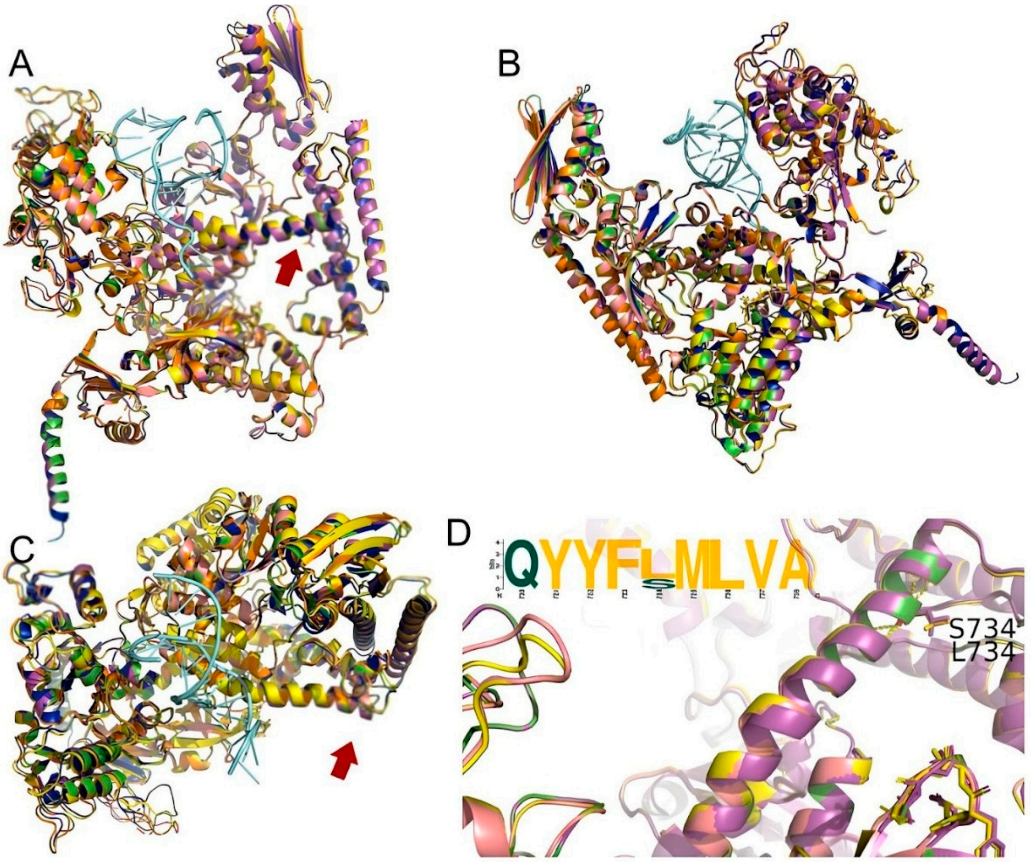

Supplementary Figure S2 - Cartoon representation of the position of the mutation in the DNA-dependent RNA polymerase gene. A) Front view of the molecule, with a red arrow pointing to the site of variation. B) Back view of the molecule. C) Up view of the molecule, with a red arrow pointing to the site of variation. D) A zoom view of the bridge helix of the cleft. The variant amino acids are shown as sticks, with the code for the variation next to them. The box in the upper left corner represents the amino acids surrounding the site of variation. Yellow letters - amino acids with a hydrophobic side chain; green letters - amino acids with an uncharged polar side chain. In the cartoons, orange - VACV DNA-dependent RNA polymerase Chain A (PDB Code 6rid); dark blue - VACV RNA polymerase Chain A (PDB code 8p0k); green - MPXV Clade II reference; light red - Clone 1; purple - Clone 2; yellow - Clone 3.

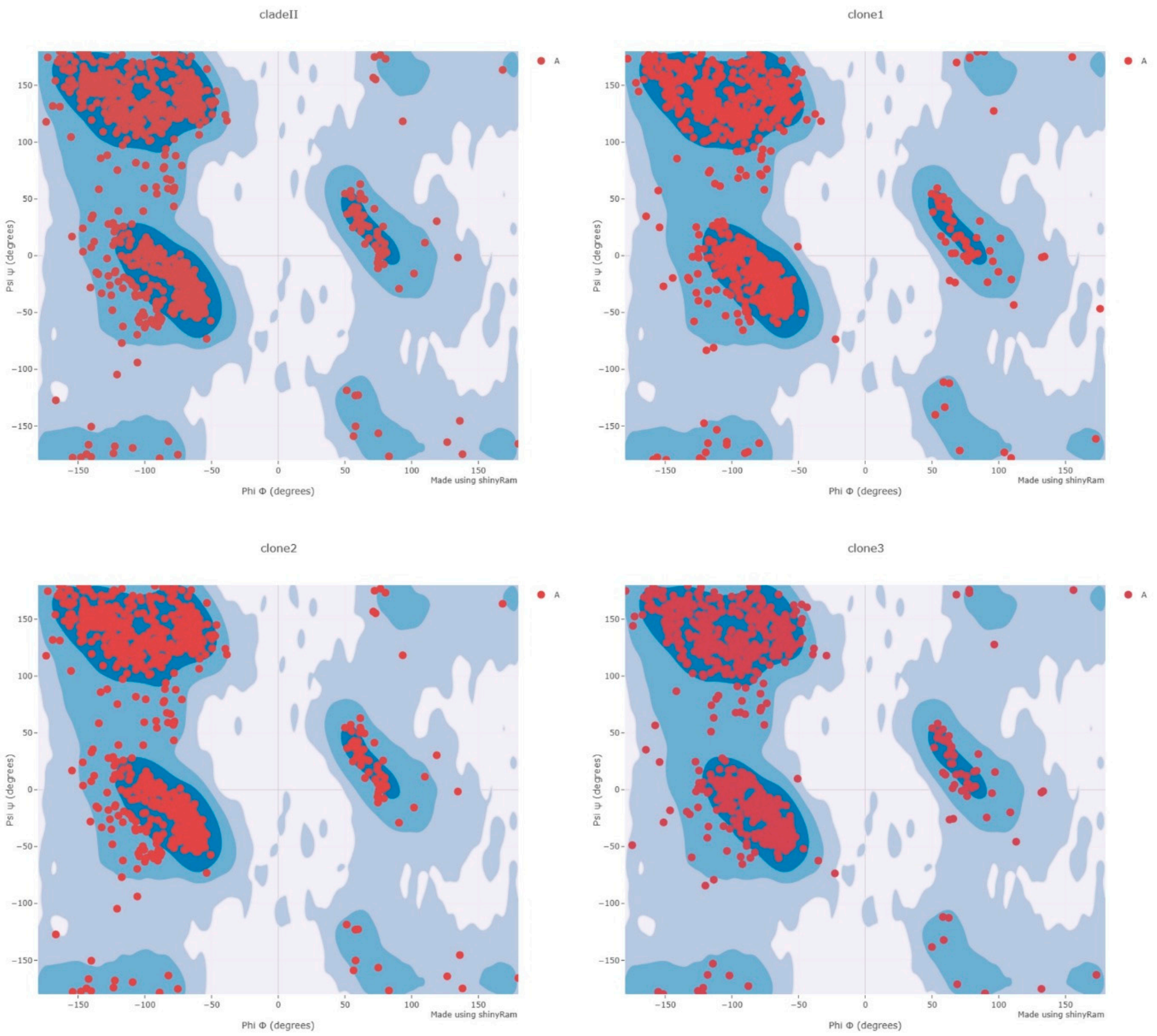

Supplementary Figure S3 - Ramachandran plots of Clade II, Clone 1, Clone 2 and Clone 3 DNA-dependent RNA polymerase models, in sequence.

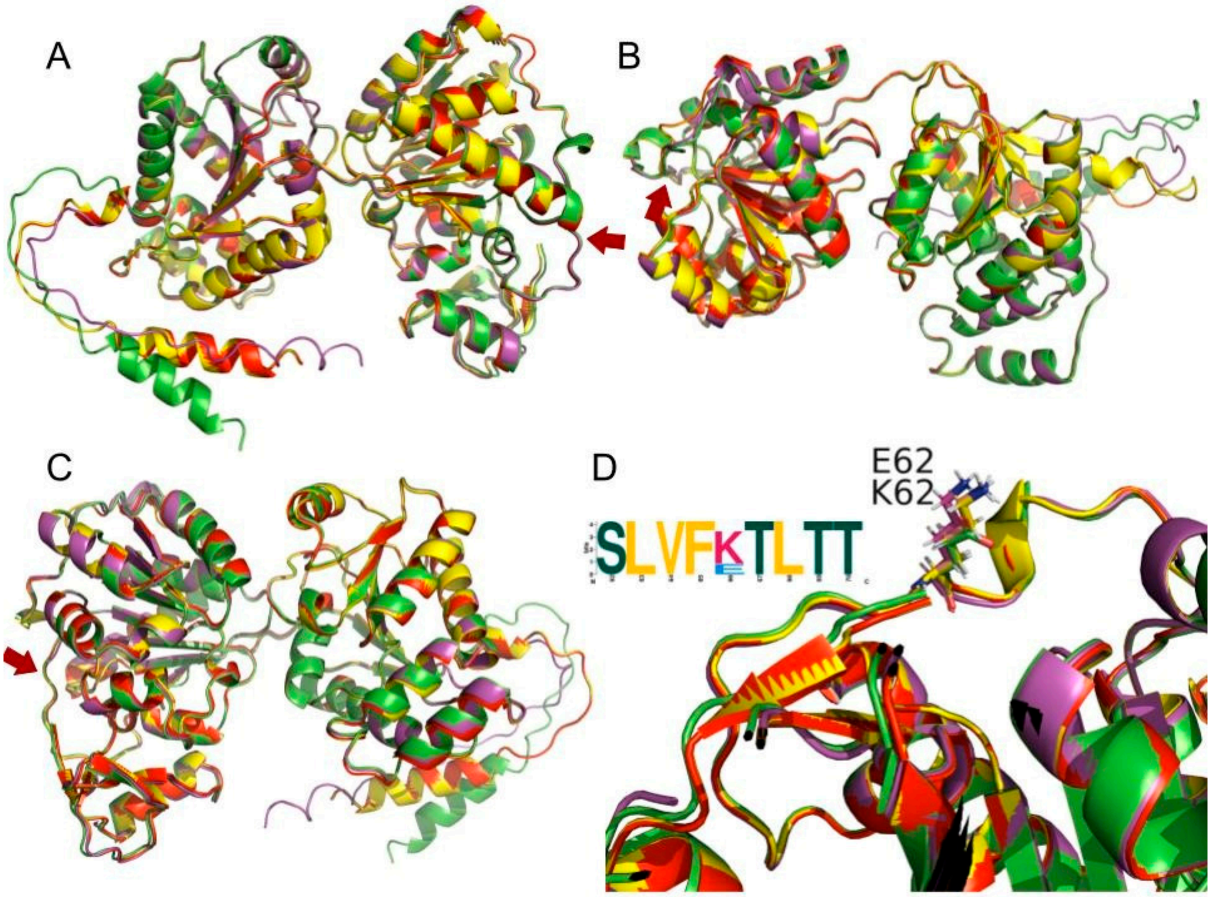

Supplementary Figure S4 - Cartoon representation of the position of the mutation in the DNA helicase gene. A) Front view of the molecule, with a red arrow pointing to the site of variation. B) Back view of the molecule, with a red arrow pointing to the site of variation. C) Up view of the molecule, with a red arrow pointing to the site of variation. D) A zoom view of disorded region in which the variation was found. The variant amino acids are shown as sticks, with the code for the variation next to them. The box in the upper left corner represents the amino acids surrounding the site of variation. Yellow letters - amino acids with a hydrophobic side chain; green letters - amino acids with an uncharged polar side chain. In the cartoons, green - MPXV Clade II reference; light red - Clone 1; purple - Clone 2; yellow - Clone 3.

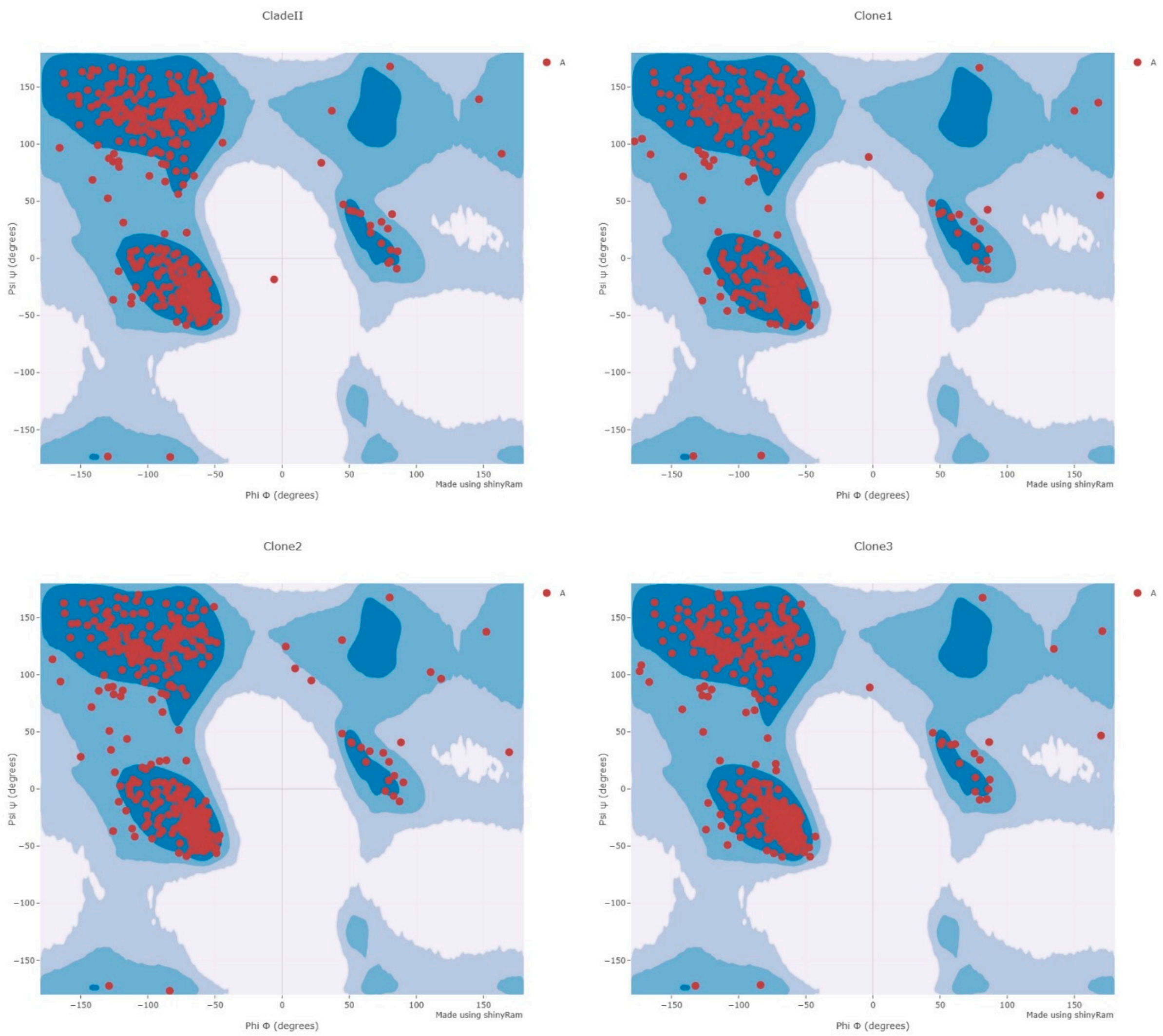

Supplementary Figure S5 - Ramachandran plots of Clade II, Clone 1, Clone 2 and Clone 3 DNA helicase models, in sequence.
